# Evaluation of AlphaFold3 for Predicting Human Heme-Binding Protein Structures

**DOI:** 10.3390/ijms27146278

**Published:** 2026-07-14

**Authors:** Ki Hyun Nam

**Affiliations:** College of General Education, Kookmin University, Seoul 02707, Republic of Korea; structure@kookmin.ac.kr

**Keywords:** AlphaFold3, heme-binding protein, heme, structure prediction, ligand docking, protein–ligand interaction

## Abstract

Heme is a crucial cofactor involved in various biological processes, such as electron transport, catalysis, and oxygen binding. Understanding the binding of heme to heme-binding proteins (HBPs) is essential for clarifying their functions and molecular mechanisms and for applications in enzyme engineering and therapeutic development. AlphaFold3 (AF3), an artificial intelligence (AI)-based macromolecular prediction tool, has been applied to the structural modeling of various proteins, including HBPs. Nevertheless, whether AF3 provides reliable structural information for HBPs has not yet been investigated. To determine the AF3 predictions for HBPs, the apo and holo states of four human HBPs, including the cytochrome b5 domain of sulfite oxidase (SO-b5), the cytochrome b5 domain of NADH cytochrome b5 oxidoreductase (Ncb5or-b5), cytochrome b5 type B (CYB5B), and neuroglobin (NGB), generated by AF3, were examined and compared with experimental HBP structures. The overall positions of the heme molecules were well docked into the heme-binding pockets of HBPs; however, there were differences in the heme-binding configurations, including pocket geometry and coordination environment, which are crucial for functional interpretation. The experimental NGB structure contains a disulfide bond near the heme-binding region, whereas the AF3-predicted model lacks this bond, causing differences in local folding that affect the heme-binding environment. Molecular dynamics simulations demonstrated that the AF3-predicted NGB structure exhibited distinct molecular conformations and flexibility compared with the experimental structure. These data indicate both the potential and the limitations of using AF3-predicted structures to model the heme-binding states of HBPs.

## 1. Introduction

Heme, an iron-containing porphyrin, is synthesized and used by a wide range of living organisms, from bacteria to humans [1,2]. It serves as a key cofactor in a wide variety of proteins, including hemoglobin, cytochromes, catalases, and peroxidases, where it plays vital roles in oxygen transport, electron transfer, and redox reactions [3,4,5,6,7]. Heme-binding proteins (HBPs) are used across a wide range of applications in life sciences, medicine, environmental technology, and industry [8,9]. For instance, cytochrome P450 (P450 or CYP) catalyzes the metabolism of various drugs and endogenous compounds, making it an important target in drug development [10,11]. Furthermore, myoglobin and hemoglobin serve as oxygen sensors or are applied in biosensor technologies [12]. In addition, haptoglobin and hemopexin are used as blood biomarkers to diagnose hemolytic disorders and oxidative stress [12,13,14]. Consequently, to improve the industrially relevant activity and stability of HBPs, various HBPs have been rationally engineered on the basis of their structural information [15,16,17]. For instance, the natural electron transfer protein cytochrome b562 was rationally redesigned by modifying its heme-binding residues to enable catalytic hydrogen production, resulting in efficient hydrogen production even in the presence of oxygen [18]. Moreover, cytochrome P450 BM-3 was engineered to improve its industrial applicability as an oxidizing agent, specifically to regioselectively and enantioselectively hydroxylate linear alkanes at targeted positions [16].

Various HBP structures have been determined to understand their molecular mechanisms and gain insights into engineering strategies to improve their activity [19,20,21,22,23]. Although experimental HBP structures provide valuable insights into heme–protein interactions, several HBPs lack experimentally resolved structures. To address this limitation, the structural information of these HBPs is frequently inferred from computationally predicted models [24,25,26,27]. Nonetheless, the reliability of the structural information of HBPs, including the heme coordination, remains uncertain.

Artificial intelligence (AI)–based prediction of protein structure, such as AlphaFold2 (AF2) [28], ESMFold [29], and RoseTTAfold [30], has opened new avenues for protein structure modeling with high accuracy. In particular, AlphaFold3 (AF3) demonstrates significantly higher accuracy in predicting protein–ligand interactions than other state-of-the-art docking tools [31]. AF3 has been widely used for understanding protein functions and facilitating applications in biotechnology [31,32,33]. AF3-predicted models have also been widely used as search models for molecular replacement in X-ray crystallography and as initial models for structure determination in cryogenic electron microscopy studies [34,35,36]. Thus, AF3 has significantly advanced various research fields; nevertheless, its predictions are not always reliable and cannot entirely replace experimental structures [37]. For instance, in fluorescent proteins, tripeptides form a chromophore via posttranslational modification, which AF3 fails to predict [38]. Fatty acid docking to fatty acid-binding proteins (FABPs) using AF3 was similar to experimental structures for FABP1 and FABP3, whereas the predicted docking results for FABP4 and FABP5 were inconsistent with experimentally determined structures [39]. Furthermore, the AF3-predicted substrate-binding cleft of the xylanase HviGH11 differed from that observed in the crystal structure, which subsequently affected the substrate docking results [40]. Hence, it is essential to determine the accuracy of AF3-predicted structures for target proteins; however, such evaluations have not yet been systematically explored for HBPs.

To evaluate the accuracy of AF3 in predicting HBP structures, the AF3-predicted apo and holo states of four human HBPs, including the cytochrome b5 domain of sulfite oxidase (SO-b5), the cytochrome b5 domain of NADH cytochrome b5 oxidoreductase (Ncb5or-b5), cytochrome b5 type B (CYB5B), and neuroglobin (NGB), were examined. The overall fold, heme-binding cavities, and heme coordination geometries of the AF3-predicted HBPs were comprehensively analyzed and compared with their experimentally determined structures. Molecular dynamics simulations were conducted for AF3-predicted and experimental NGB structures, which displayed different protein folding. This study provides a comprehensive evaluation of the utility and limitations of AF3 in modeling HBP structures and heme coordination, thus offering insights for future applications in HBP functional analysis and enzyme engineering.

## 2. Results

### 2.1. AF3-Predicted HBPs

To determine the accuracy of AF3 in predicting HBPs, structural comparisons between AF3-predicted and experimentally determined HBP structures were crucial. To date, 1201 heme-bound human HBP structures are available in the Protein Data Bank (PDB). Based on the criteria of resolution (<1.5 Å), R_free_ (<25%), and overall B-factor (<30 Å^2^), 24 HBP structures from seven unique human HBPs, including sulfite oxidase, cytochrome b5 reductase 4, cytochrome b5 type B, cytochrome P45011β, cholesterol 24-hydroxylase, hemoglobin, and neuroglobin, were filtered. Among them, the crystal structures of cytochrome P450 11β were complexed with fadrozole (PDB code: 6M7X, residues 31–503) [41] or metyrapone (PDB code: 7E7F, residues 28–503) [42], and the crystal structure of cholesterol 24-hydroxylase (CYP46A1, Ser28–Ala494) was complexed with compound 3f. All these compounds were located on the heme molecule and interacted with the iron atom in the heme, which can affect the geometry of the heme molecules in the AF3-predicted structure. Furthermore, because hemoglobin forms a tetrameric assembly composed of four subunits, its structure may be affected not only by heme binding but also by oligomeric interactions. Accordingly, AF3 prediction evaluation for these three HBPs was excluded from this study. Therefore, four HBPs, including the cytochrome b5 domain of sulfite oxidase (SO-b5), the cytochrome b5 domain of NADH cytochrome b5 oxidoreductase (Ncb5or-b5), cytochrome b5 type B (CYB5B), and neuroglobin (NGB), were selected as target proteins to determine the accuracy of AF3 predictions (Table S1).

**Figure 1:**
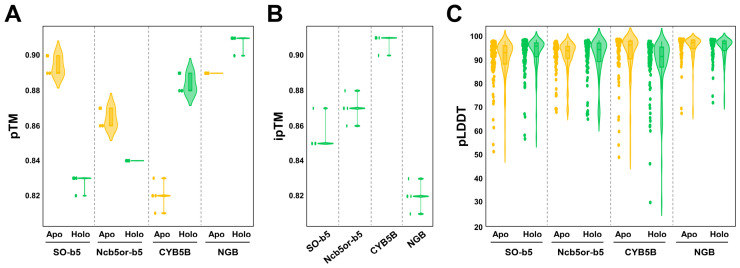
Analysis of the confidence score of AF3-predicted apo and holo states of HBP structures. Violin plots of (**A**) pTM, (**B**) ipTM, and (**C**) pLDDT values for the AF3-predicted apo (yellow) and holo (green) state structures of SO-b5, Ncb5or-b5, CYB5B, and NGB.

The conformation of the heme-binding site in HBPs depends on the apo or heme-bound (holo) state of the protein [43]. To determine whether AF3 can capture these structural differences, the apo and holo forms of Hb, SO-b5, Ncb5or-b5, CYB5B, and NGB predicted by AF3 were compared. The AF3 server generated five structural models for each HBP, and the quality of these models was examined using confidence metrics, including the predicted Template Modeling score (pTM) and the interface predicted Template Modeling score (ipTM) [31]. A pTM score of >0.5 suggests that the overall predicted fold resembles the true structure, whereas an ipTM score of >0.8 indicates high-confidence predictions of subunit interfaces. The pTM values for the apo and holo states of SO-b5, Ncb5or-b5, CYB5B, and NGB were 0.82–0.89 and 0.81–0.91, respectively (Figure 1A). The ipTM values for the holo states of SO-b5, Ncb5or-b5, and NGB were 0.85–0.88, whereas those for CYB5B were relatively lower, ranging from 0.75 to 0.78 (Figure 1B). The average predicted local distance difference test (pLDDT) values for the highest-scoring AF3 models of SO-b5, CYB5B, and NGB were 90.82–95.14 and 91.31–95.49 for the apo and holo states, respectively. Meanwhile, the pLDDT values for the apo and holo states of Ncb5or-b5 were 87.96 and 92.34, respectively (Figure 1C). These data indicate that most AF3-predicted HBP structures are reliable according to AF3 confidence criteria, although relatively lower ipTM and pLDDT values were observed for CYB5B and Ncb5or-b5, respectively.

### 2.2. Structural Comparison Between AF3-Predicted and Experimental HBP Structures

#### 2.2.1. Cytochrome b5 Heme-Binding Domain of Sulfite Oxidase

Sulfite oxidase (SO) is a heme-containing molybdoenzyme involved in sulfate metabolism [44,45]. It catalyzes the final step in the catabolism of the sulfur-containing amino acids cysteine and methionine and plays a crucial role in detoxifying sulfite derived from both metabolic and environmental sources [44,45]. The human SO comprises 545 amino acids and forms a homodimer. Moreover, the human SO consists of three domains: cytochrome b5 heme-binding domain, Moco domain, and an immunoglobin-like domain involved in the homodimerization of SO [46]. To date, only the crystal structure of the cytochrome b5 heme-binding domain of SO (termed SO-b5) has been determined [47]. SO-b5 consists of six α-helices and four β-strands, forming the mixed α + β architecture. The β-sheet, along with five α-helices, forms a heme-binding cavity. In the crystal structures of SO-b5, the B-factors of the heme molecules and protein were determined as 10.43 and 13.42 Å^2^, respectively, indicating that the heme molecules are rigidly bound to the HBPs.

**Figure 2:**
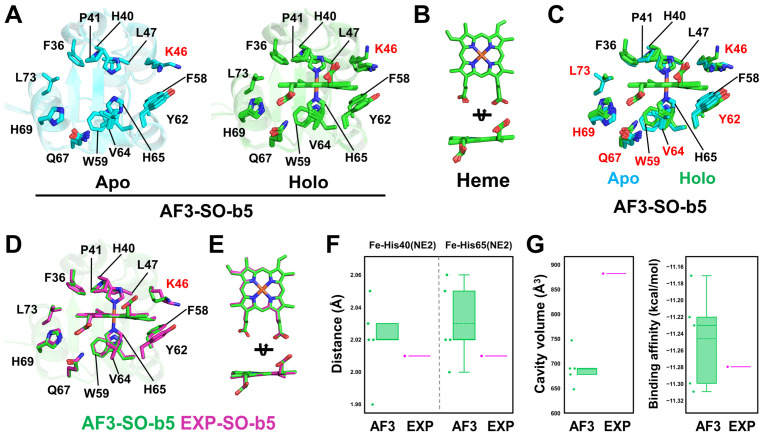
Structural analysis of the AF3-predicted SO-b5 structure. (**A**) Superimposition of the heme-binding site in the apo and holo states of AF3-SO-b5. (**B**) The heme molecules in the superimposed holo state of AF3-SO-b5. (**C**) Superimposition of the apo (cyan) and holo (green) states of AF3-SO-b5. (**D**) Superimposition of the holo state of AF3-SO-b5 (green) with the crystal structure of SO-b5 (purple, PDB code: 1MJ4). (**E**) Heme molecules in the superimposed holo-state structures of AF3-SO-b5 (green) and EXP-SO-b5 (purple). (**F**) Interaction distances between the Fe ion of heme and histidine residues in SO-b5 from AF3 (green) and experimental (purple) structures. (**G**) Calculated heme-binding cavity volume and binding affinity between heme and protein in AF3-predicted (green) and experimental (purple) SO-b5 structures.

AF3 generated the five model structures for each apo and holo state of the HBP models. Superimposition of the apo-state models of AF3-SO-b5 yielded RMSD values of 0.051–0.078 Å, and superimposition of the holo-state models yielded RMSD values of 0.068–0.134. Å (Figure 2A and Figure S1). In the superimposition of the apo and holo states of the AF3-SO-b5 models, the conformations of residues involved in heme interaction were highly similar, with the exception of subtle movements in Lys46, Val64, and Gln67 (Figure 2A). The positions of the heme molecules in the AF3-SO-b5 structures were also highly consistent, except for minor variations in the propionate groups (Figure 2B).

Next, the AF3 models for each state were compared to determine the structural differences between the apo and holo states of AF3-HBPs. Superimposition of the apo and holo states of the AF3-SO-b5 structures yielded an RMSD value of 0.427 Å, indicating a slight difference in conformation in the unstructured N-terminal region (Supplementary Figure S2). In the superimposition of the apo and holo states of AF3-SO-b5, the side chains of the Phe58 and Tyr62 residues of AF3-SO-b5 shifted to the heme-binding pocket, whereas the Trp59, Val64, His69, and Leu73 residues shifted away from the heme-binding pocket, compared with that in the superimposition of the apo state of AF3-SO-b5 (Figure 2C). The distance between the NE2 atoms of two histidine (His40 and His65) residues coordinating the heme iron was 3.82 Å in the apo state and 4.03 Å in the holo state, indicating that the heme-binding pocket in the holo state was slightly wider.

Superimposition of the holo state of AF3-SO-b5 with experimentally determined SO-b5 (EXP-SO-b5) yielded RMSD values of 0.165–0.217 Å (Figure 2D and Figure S3). The heme molecule bound to AF3-SO-b5 displayed an almost identical position and conformation to that in EXP-SO-b5, exhibiting only subtle positional differences within 0.4 Å (Figure 2E), indicating that the heme-binding configuration is highly consistent. Nonetheless, the detailed interactions between the heme molecule and SO-b5 differed.

The superimposition of AF3-SO-b5 and EXP-SO-b5 demonstrated that the positions and conformations of the residues (Phe36, Leu47, and L73) located inside the heme-binding pocket were almost identical (Figure 2D). In contrast, the residues (Pro41, Phe58, Val64, Tyr62, and His69) located in the solvent-exposed region exhibited subtle positional shifts in their side chains (Figure 2D). The side-chain conformation of Lys46, which is involved in hydrophobic interactions with heme, also differed between the AF3-predicted and experimental structures (Figure 2D).

In AF3-SO-b5, the iron atom of the heme was coordinated by His40 and His65 at distances of 1.98–2.05 and 2.00–2.06 Å, respectively, whereas in the EXP-SO-b5 structure, the iron atom of the heme was coordinated by the NE2 atoms of His40 and His65, both at a distance of 2.01 Å (Figure 2F). Moreover, other interactions between the heme molecule and SO-b5 exhibited slight differences between the AF3-predicted models and experimental structures (Figure 2F). The average cavity volumes of the heme-binding pocket were 648–747 Å^3^ for AF3-SO-b5 and 882 Å^3^ for EXP-SO-b5 (Figure 2G). The calculated heme-binding affinities (ΔG) were 11.17–11.31 kcal/mol for AF3-SO-b5 and 11.28 kcal/mol for EXP-SO-b5 (Figure 2G), indicating that subtle movements of the residues located near the heme molecule influence the structural properties.

#### 2.2.2. NADH Cytochrome b5 Oxidoreductase

NADH cytochrome b5 oxidoreductase (Ncb5or; also known as Cyb5R4, b5/b5R, or b5 + b5R) is an important enzyme involved in metabolic processes and serves as a vital component of the electron transfer system [48]. Ncb5or is expressed in a wide range of tissues and cells in animals. The human Ncb5or consists of 521 amino acids and three structural domains: the N-terminal cytochrome b5 heme-binding domain (Ncb5or-b5, residues 54–130) and the C-terminal FAD-binding FR-type b5R domain (Ncb5or-b5R, residues 273–385), which are linked by the CS domain (residues 165–256) [49]. To date, only the domain structures of CYB5R4 have been determined as follows: the Ncb5or-b5 domain (PDB code: 3LF5, residues 51–137) [49] and the Ncb5or-b5R domain (PDB codes: 6MV1 and 6MV2, residues 164–521). The Ncb5or-b5 domain comprises five α-helices and four β-strands, forming a mixed α + β architecture. The β-sheet, along with four α-helices, forms a heme-binding cavity. The heme-bound Ncb5or-b5 contains two molecules in the asymmetric unit and demonstrates high similarity, with an RMSD value of 0.144 Å. Remarkably, two distinct heme-binding poses were identified in the heme-binding cavity of Ncb5or-b5, differing by an ~180° rotation around the porphyrin macrocycle axis (Supplementary Figure S4). Superimposition of the two Ncb5or-b5 molecules revealed that the position of the hydrophobic porphyrin core of heme exhibited subtle movement, and the propionate side chains adopted various conformations (Supplementary Figure S4). The position of the iron atom in the heme differed by 0.1 Å between the two Ncb5or-b5 molecules. In the crystal structure of Ncb5or-b5, the B-factors of the heme molecule and protein were 10.95 and 15.24 Å^2^, respectively, indicating that the heme molecules are rigidly bound to the HBPs.

**Figure 3:**
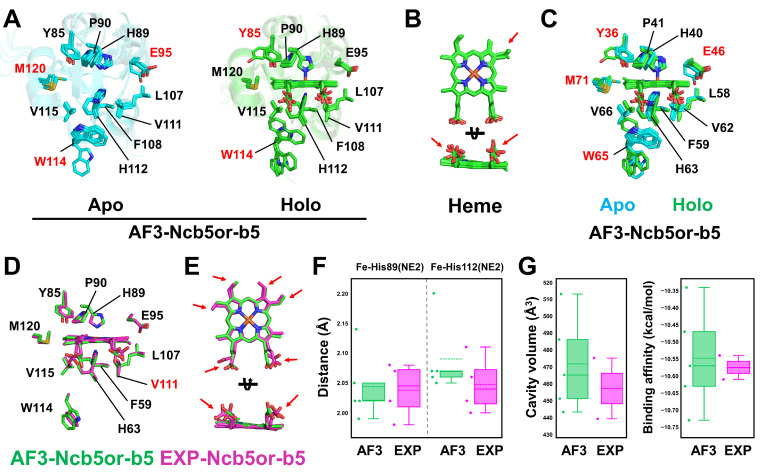
Structural analysis of the AF3-predicted Ncb5or-b5 structure. (**A**) Superimposition of the heme-binding site in the apo and holo states of AF3-Ncb5or-b5. (**B**) Close-up view of the heme molecules in the superimposed holo state of AF3-Ncb5or-b5. (**C**) Superimposition of the apo (yellow) and holo (green) states of AF3-Ncb5or-b5. (**D**) Superimposition of the holo state of AF3-Ncb5or-b5 (green) with the experimental Ncb5or-b5 structure (purple, PDB code: 3LF5). (**E**) Heme molecules in the superimposed holo structures of AF3-Ncb5or-b5 (green) and EXP-Ncb5or-b5 (purple). (**F**) Interaction distances between the Fe ion of heme and histidine residues in Ncb5or-b5 from AF3-predicted (green) and experimental (purple) structures. (**G**) Calculated heme-binding cavity volume and binding affinity between heme and protein in AF3-predicted (green) and experimental (purple) Ncb5or-b5 structures.

Superimposition of the apo-state models of AF3-Ncb5or-b5 yielded RMSD values of 0.105–0.301 Å, whereas the superimposition of the holo-state models yielded RMSD values of 0.183–0.271 Å, with differences observed in the conformations of the N- and C-terminal unstructured regions (Figure 3A and Figure S1). In the holo state of AF3-Ncb5or-b5, the positions of the porphyrin rings of the heme molecules were highly similar; however, subtle differences existed in the orientations of the propionate and vinyl groups (Figure 3B).

Superimposition of the apo and holo states of the AF3-Ncb5or-b5 structures yielded an RMSD value of 0.418 Å, indicating a significantly different conformation in the unstructured N- and C-terminal regions (Figure 3C and Figure S2). In the superimposition of the apo and holo states of AF3-Ncb5or-b5, most side-chain conformations of the residues involved in forming the heme-binding pocket were identical; however, their positions shifted due to differences in the main chain positions between the apo and holo states of AF3-Ncb5or-b5 (Figure 3C). In particular, the NE2 atoms of two histidine residues (His40 and His63) coordinating the heme iron exhibited significantly different positions in the heme-binding cavity. The distance between these two histidine residues was 4.40 Å in the apo state and 4.33 Å in the holo state, indicating that the heme-binding pocket in the holo state was slightly narrowed.

Superimposition of the holo state of AF3-Ncb5or-b5 with EXP-Ncb5or-b5 yielded RMSD values of 0.131–0.286 Å (Figure 3D and Figure S3). Structural superimposition of AF3-Ncb5or-b5 and EXP-Ncb5or-b5 demonstrated that the positions and conformations of four hydrophobic residues (Phe59, Pro90, Val115, and Met120) located within the heme-binding pocket were almost identical, whereas a positional shift in the side chain of Tyr85 was detected (Figure 3D). In EXP-Ncb5or-b5, the CG1 and CG2 atoms of the heme-interacting Val111 side chain pointed toward the heme molecule, whereas in AF3-Ncb5or-b5, the CG1 atom was directed toward the heme, but the CG2 atom pointed in the opposite direction.

The position of the heme molecule within the heme-binding pocket slightly differed between AF3-Ncb5or-b5 and EXP-Ncb5or-b5, with the maximum deviation of the iron atom in the heme being 0.28 Å. Moreover, the conformation or position of the vinyl, methyl, and propionate groups of the heme differed between the AF3-Ncb5or-b5 and EXP-Ncb5or-b5 structures (Figure 3E). These propionate groups of the heme molecule did not interact with the protein and may adopt diverse conformations, as found in the experimental structure.

In the AF3-Ncb5or-b5 structure, the iron atom is coordinated by the NE2 atoms of His89 and His112 at distances of 1.99–2.14 and 2.05–2.20 Å, respectively (Figure 3F). In the EXP-Ncb5or-b5 structure, the iron atom of the heme is coordinated by the NE2 atoms of His89 and His112, at distances of 1.98–2.08 and 2.02–2.06 Å, respectively (Figure 3F).

The average cavity volumes of the heme-binding pocket were 443–513 Å^3^ for AF3-Ncb5or-b5 and 439 and 475 Å^3^ for EXP-Ncb5or-b5 (Figure 2G). The calculated heme-binding affinities (ΔG) were 10.34–10.73 kcal/mol for AF3-Ncb5or-b5 and 11.54 and 11.61 kcal/mol for EXP-Ncb5or-b5 (Figure 2G).

#### 2.2.3. Cytochrome b5 Type B

Cytochrome b5 is a membrane-bound hemoprotein that functions as an electron donor and delivers reducing power to the terminal enzymes involved in oxidative reactions [50,51]. The human cytochrome b_5_ type B (CYB5B) consists of 150 amino acids, with the N-terminal 20 residues predicted to form a disordered region. The crystal structure of CYB5B, excluding this disordered region, has been determined (PDB code: 3NER; residues 16–107) [52]. CYB5B consists of six α-helices and five β-strands, forming a mixed α + β architecture. The β-sheet, along with five helices, forms a heme-binding cavity. The heme-bound CYB5B contains two molecules in the asymmetric unit and demonstrates similarity with an RMSD value of 0.271 Å. Superimposition of the two CYB5B molecules revealed that the position of the hydrophobic porphyrin core of the heme exhibited a subtle shift of 0.65 Å, and the propionate groups in the heme molecule adopted various conformations (Supplementary Figure S5). These findings suggest the variability of heme binding in CYB5B. In the crystal structure of CYB5B, the B-factor values of the heme molecule and protein were determined as 16.85 and 18.92 Å^2^, respectively, indicating that the heme molecules are rigidly bound to the HBPs.

**Figure 4:**
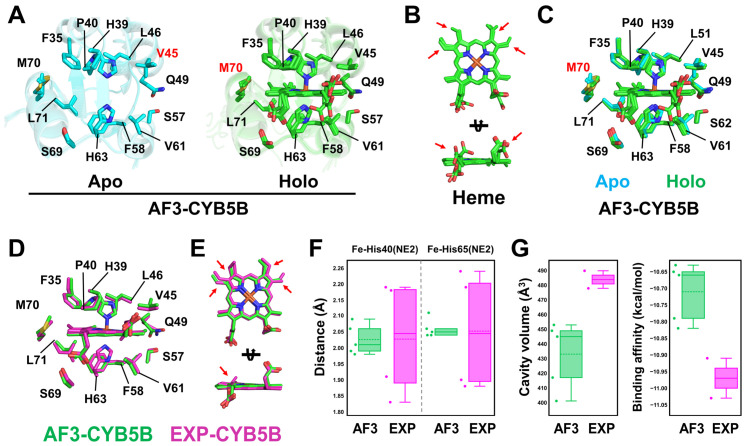
Structural analysis of the AF3-predicted CYB5B structure. (**A**) Superimposition of the heme-binding site in the apo and holo states of AF3-CYB5B. (**B**) Close-up view of the heme molecules in the superimposed holo state of AF3-CYB5B. (**C**) Superimposition of the apo (yellow) and holo (green) states of AF3-CYB5B. (**D**) Superimposition of the holo state of AF3-CYB5B (green) with the experimental CYB5B structure (purple, PDB code: 3NER). (**E**) Heme molecules in the superimposed holo structures of AF3-CYB5B (green) and EXP-CYB5B (purple). Residues and arrows in red indicate conformational differences. (**F**) Interaction distances between the Fe ion of heme and histidine residues in CYB5B from AF3-predicted (green) and experimental (purple) structures. (**G**) Calculated heme-binding cavity volume and binding affinity between heme and protein in AF3-predicted (green) and experimental (purple) CYB5B structures.

Superimposition of the apo-state models of AF3-CYB5B yielded RMSD values of 0.075–0.154 Å, whereas the superimposition of the holo-state models yielded RMSD values of 0.116–0.354 Å, with differences found in the conformations of the N-terminal unstructured regions (Figure 4A and Figure S1). In the holo state of AF3-CYB5B, the positions of the porphyrin rings were highly conserved; however, a flipped orientation of the heme (~180° rotation) was detected in five molecules within the asymmetric unit, resulting in altered positions of the vinyl and methyl substituents. Furthermore, conformational variability and positional shifts in the propionate groups were detected (Figure 4B).

Superimposition of the apo and holo states of the AF3-CYB5B structure yielded an RMSD value of 0.372 Å, indicating a significantly different conformation in the unstructured N-terminal region (Supplementary Figure S3). In the superimposition of the apo and holo states of AF3-CYB5B, the position and conformation of most residues involved in forming the heme-binding pocket were highly similar, except for conformational differences in the side chains of Val50, Ser69, and Met75 (Figure 4C). The position of His44, which coordinates the heme iron, was almost identical, whereas His68, which also coordinates the heme iron, was displaced by 0.4 Å. The distance between these two histidine residues was 4.20 Å in the apo state and 4.07 Å in the holo state, indicating a slight narrowing of the heme-binding pocket in the holo state.

The superimposition of AF3-CYB5B and EXP-CYB5B yielded RMSD values of 0.298–0.528 Å (Supplementary Figure S3). The conformations of the residues (Phe35, Pro40, His39, Val45, Leu46, Gln49, Ser57, Phe58, Val61, His63, Ser69, Met70, and Leu71) located near the heme molecule in AF3-CYB5B and EXP-CYB5B were almost identical, whereas subtle movements of the side chains were detected within 0.9 Å (Figure 4D). The position of the heme molecule and the conformations of its vinyl, methyl, and propionate groups within the heme-binding pocket differed between AF3-CYB5B and EXP-CYB5B, probably due to the intrinsic flexibility of the heme observed in the experimental structure (Figure 4E).

In AF3-CYB5B, the iron atom of the heme was coordinated by the NE2 atoms of His39 and His63 at distances of 1.98–2.09 and 2.04–2.11 Å, respectively (Figure 4F). In EXP-CYB5B, the iron atom of the heme was coordinated by the NE2 atoms of His39 and His63 at distances of 1.83–2.19 and 1.88–2.24 Å, respectively (Figure 4F). The average cavity volumes of the heme-binding pocket were 478 and 490 Å^3^ for EXP-CYB5B and 401–453 Å^3^ for AF3-CYB5B (Figure 4G). The calculated heme-binding affinities (ΔG) were 10.91 and 11.03 kcal/mol for EXP-CYB5B and 10.63–10.82 kcal/mol for AF3-CYB5B (Figure 4G).

#### 2.2.4. Neuroglobin

NGB is a member of the globin family predominantly expressed in brain neurons [53]. It exhibits oxygen-binding capacity and improves cell viability under hypoxia and various types of oxidative stress in transgenic systems [54]. The crystal structure of the human NGB-A15C mutant (PDB code: 7VQG) was determined, where the mutation site is located on the helix bundle, with a distance of 22.50 Å between the Cα atom of Cys15 and the iron atom in the heme, and does not interact with the heme. NGB consists of eight α-helices that form an α-helix bundle, with four of these helices constituting the heme-binding cavity. In the crystal structure of NGB, the B-factors of the heme molecules and proteins were 19.64 and 22.14 Å^2^, respectively, indicating that the heme molecules are rigidly bound to the HBPs.

**Figure 5:**
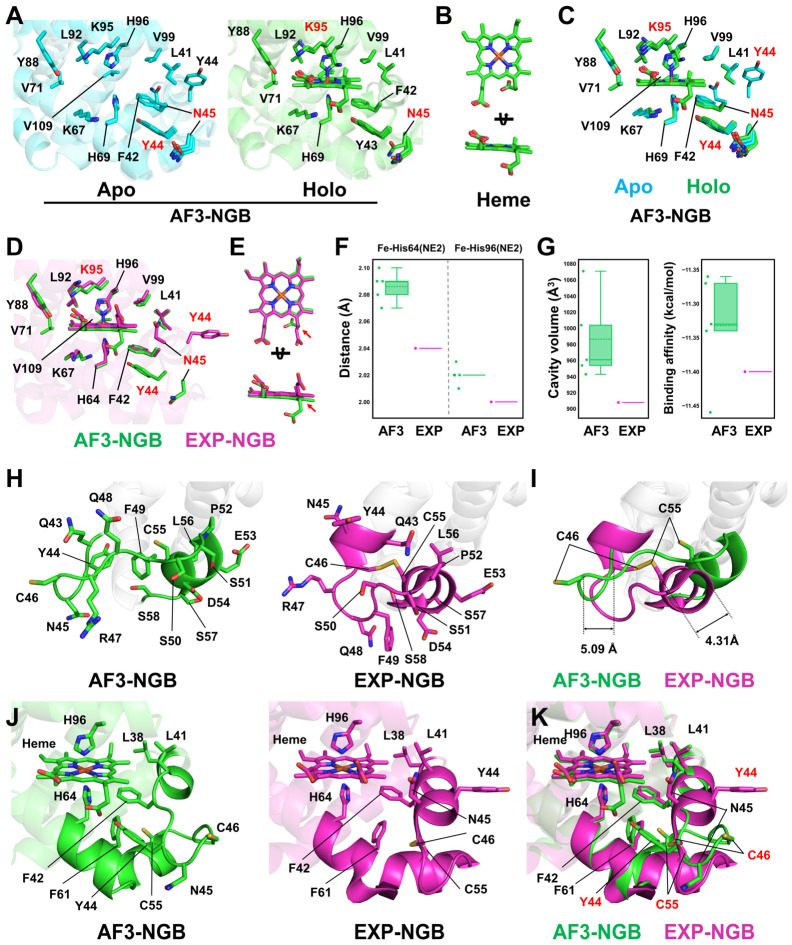
Structural analysis of the AF3-predicted NGB structure. (**A**) Superimposition of the heme-binding site in the apo and holo states of AF3-NGB. (**B**) Close-up view of the heme molecules in the superimposed holo state of AF3-NGB. (**C**) Superimposition of the apo (yellow) and holo (green) states of AF3-NGB. (**D**) Superimposition of the holo state of AF3-NGB (green) with the experimental NGB structure (purple, PDB code: 7VQG). (**E**) Heme molecules in the superimposed holo structures of AF3-NGB (green) and EXP-NGB (purple). Residues and arrows in red indicate conformational differences. (**F**) Interaction distances between the Fe ion of heme and histidine residues in NGB from AF3-predicted (green) and experimental (purple) structures. (**G**) Calculated heme-binding cavity volume and binding affinity between heme and protein in AF3-predicted (green) and experimental (purple) NGB structures. (**H**) Close-up view of the different folding between AF3-NGB and EXP-NGB depending on disulfide bond formation. (**I**) Superimposition of the folding regions in AF3-NGB and EXP-NGB. (**J**) Close-up view of the heme environment in AF3-NGB and EXP-NGB. (**K**) Superimposition of the heme-binding environments of AF3-NGB and EXP-NGB.

Superimposition of the apo-state models of AF3-NGB yielded RMSD values of 0.059–0.133 Å, whereas the superimposition of the holo-state models yielded RMSD values of 0.074–0.209 Å (Figure 5A and Figure S1). The residues located near the heme molecule demonstrated almost identical conformations with subtle movements; however, the position of one Asn45 residue in the apo state of AF3-NGB differed from that of the other four Asn45 residues (Figure 5A). The positions of the porphyrin rings were highly conserved, and only subtle movements of the propionate groups were detected (Figure 5B). Superimposition of the apo and holo states of the AF3-NGB structures yielded an RMSD value of 0.173 Å, indicating slight conformational differences in the N-terminus and a loop region between α-helices (Figure 5C and Figure S2). In the superimposition of the apo and holo states of AF3-NGB, the position and conformation of most residues involved in forming the heme-binding pocket were highly similar, except for a subtle movement of the Asn45 residue, which is involved in interaction with the heme molecule (Figure 5C). The position of His64, which is involved in coordinating the heme iron, was highly similar; however, the position of His96, which is also involved in iron coordination, shifted by 1.20 Å. The distance between these two histidine residues was 5.07 Å in the apo state and 4.10 Å in the holo state, indicating a slight narrowing of the heme-binding pocket in the holo state.

Superimposition of the holo state of AF3-NGB with EXP-NGB yielded RMSD values of 0.360–0.404 Å (Figure 5D and Figure S3). The positions of the residues (Leu41, Phe42, His64, Lys67, Val71, Tyr88, Leu92, His96, Val99, and Val109) located near the heme molecule were identical; however, the main-chain and side-chain conformations of Tyr44 and Asn45 differed significantly between AF3-NGB and EXP-NGB. The Cα–Cα distances for Tyr44 and Asn45 between AF3-NGB and EXP-NGB were 6.16 and 7.18 Å, respectively, probably due to the failure to form a disulfide bond in the AF3 model structure (see below).

The displacement of the heme iron between AF3-NGB and EXP-NGB was 0.37 Å (Figure 5E). Despite the different heme environments, the position of the porphyrin core within the heme-binding pocket was similar between AF3-NGB and EXP-NGB, except for conformational differences in the propionate groups (Figure 5E). In AF3-NGB, the iron atom of the heme was coordinated by the NE2 atoms of His64 and His96 at distances of 2.07–2.10 and 2.01–2.03 Å, respectively (Figure 5F). In EXP-NGB, the iron atom of the heme was coordinated by the NE2 atoms of His64 and His96 at distances of 2.04 and 2.00 Å, respectively (Figure 5F). The average cavity volumes of the heme-binding pocket were 943–1071 Å^3^ for AF3-NGB and 908 Å^3^ for EXP-NGB (Figure 5G). The calculated heme-binding affinities (ΔG) were 11.26–11.46 kcal/mol for AF3-NGB and 11.40 kcal/mol for EXP-NGB (Figure 5G).

As described earlier, the positions of Tyr44 and Asn45 and the conformations of the heme propionate groups differed significantly between AF3-NGB and EXP-NGB, which was due to differences in the folding of the Gln43–Ser59 region between the two structures (Figure 5H). Neuroglobin (NGB) contains four cysteine residues (Cys15, Cys46, Cys55, and Cys120). In both the holo state of EXP-NGB and the AF3-predicted NGB structure, Cys15 and Cys120 form a disulfide bond (Supplementary Figure S6). Meanwhile, in EXP-NGB, the residues Phe42–Cys46 form an α-helix, and Cys46 forms a disulfide bond with Cys55 located near the α-helix (Figure 5H). In contrast, in AF3-NGB, the Phe42–Cys46 region adopts a loop conformation, and the side chain of Cys46 is oriented toward the solvent, opposite to the heme molecule, and does not form a disulfide bond with Cys55 (Figure 5H), indicating the differences in protein folding. The Cα–Cα distances between Cys46 and Cys55 were 13.39 Å in AF3-NGB and 5.88 Å in EXP-NGB. The Cα–Cα distance between the Cys46 residues in AF3-NGB and EXP-NGB was 5.09 Å, whereas that between the Cys55 residues was 4.31 Å (Figure 5I). The failure of disulfide bond formation between Cys46 and Cys55 in AF3-NGB was observed in both the apo and holo states of the NGB models.

These differences in protein folding between AF3-NGB and EXP-NGB result in significant conformational changes in the α-helix spanning Pro52–Ser57, as well as in the heme environment (Figure 5I,J). In EXP-NGB, the side chain of Tyr44 is oriented toward the solvent, opposite to the heme molecule, whereas in AF3-NGB, the Tyr44 side chain faces the heme and forms hydrophobic interactions with the heme propionate group, which adopts a conformation different from that in EXP-NGB (Figure 5J). Moreover, the side chain of Asn45 in EXP-NGB is oriented toward the heme molecule, whereas in AF3-NGB, it is directed away from the heme (Figure 5J). Superimposition of the heme-binding environments of AF3-NGB and EXP-NGB revealed substantial differences involving Tyr44 and Asn45, as well as subtle side-chain movements of Leu38, Leu41, Phe42, and Phe61 located near the heme molecule (Figure 5J).

Meanwhile, the selected experimental NGB structure contains crystallization solution-derived 1,4-dioxane molecules and sulfate ions located near the heme-binding pocket and disulfide-bond region, respectively, which could potentially influence the local structure and the orientation of the heme propionate group (Supplementary Figure S7). Therefore, the structural differences observed between AF3-NGB and EXP-NGB in the heme-binding region may be influenced not only by the disulfide-bond state but also by the presence of crystallization solution-derived 1,4-dioxane and sulfate ions in the experimental structure.

Meanwhile, a ligand-free NGB structure determined at 1.74 Å resolution (PDB code: 4MPM) is available. This structure contains two NGB molecules in the asymmetric unit, both of which possess a disulfide bond between Cys46 and Cys55 (Supplementary Figure S7). Notably, the two molecules adopt different conformations in the disulfide-bond region, indicating conformational heterogeneity within the experimental NGB structure. The disulfide-bond region and the surrounding heme-binding environment of EXP-NGB still differed from those of AF3-NGB (Supplementary Figure S8). These observations suggest that AF3-NGB does not completely reflect the disulfide-bonded conformational states observed experimentally and the conformational heterogeneity present in experimental NGB structures.

### 2.3. Molecular Dynamics Simulation of NGB

Structural comparisons revealed that AF3 fails to form the disulfide bond between Cys46 and Cys55 in NGB. The disulfide bond plays a vital role in structural stability and flexibility [55]. Accordingly, the molecular dynamics of the disulfide bond region, including the heme-binding site, were expected to differ between the AF3-predicted and experimental NGB structures. To determine how potential inaccuracies in the AF3-predicted model may affect downstream interpretations, such as molecular dynamics and conformational flexibility, all-atom MD simulations of the apo and holo states of both AF3-predicted and experimental NGB were conducted at 310 K, which corresponds to the physiological temperature of the human body.

**Figure 6:**
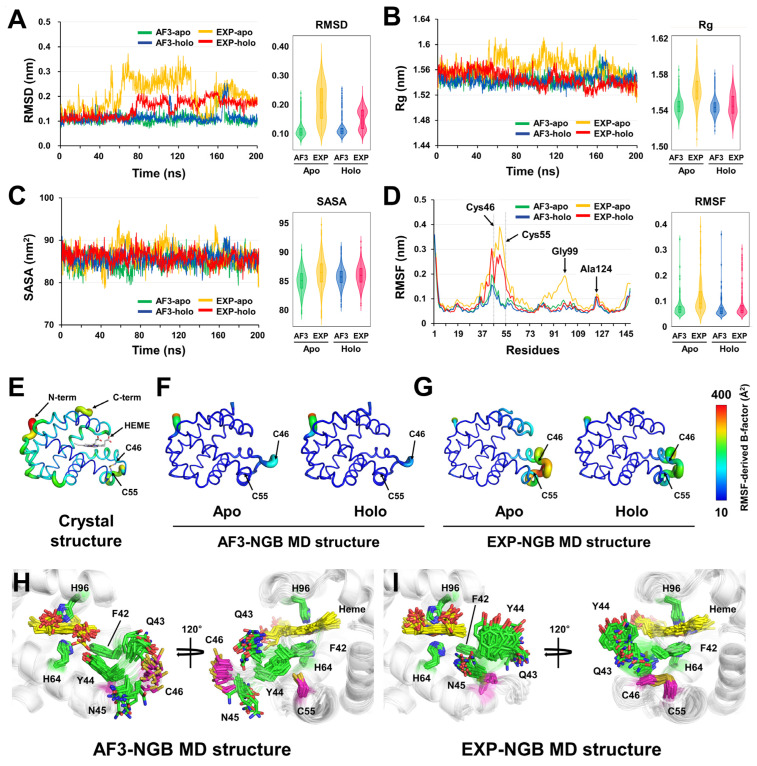
Molecular dynamics simulations of AF3-predicted and experimental NGB structures. (**A**) RMSD, (**B**) Rg, (**C**) SASA, and (**D**) RMSF values obtained from MD simulations of AF3-NGB and EXP-NGB at 310 K. (**E**) B-factor putty representation of the crystal structure of NGB (PDB code: 7VQG). RMSF-derived B-factor representations of (**F**) AF3-NGB and (**G**) EXP-NGB from MD trajectories. Dynamic ensembles of the heme-binding region associated with the disulfide bond region in (**H**) AF3-NGB and (**I**) EXP-NGB from MD trajectories at 199.8–200 ns.

The RMSD analysis demonstrated that both the apo and holo states of AF3-NGB exhibited relatively stable fluctuations throughout the simulations, except for a transient peak detected around 160–170 ns, after which the RMSD values returned to stable levels (Figure 6A). Conversely, the apo and holo states of EXP-NGB showed significant structural fluctuations during 60–190 and 70–160 ns, respectively (Figure 6A). Although both systems stabilized after 190 and 160 ns, respectively, the fluctuation levels of EXP-NGB at 200 ns remained higher than those of AF3-NGB by >1 Å. The average RMSD values of the apo and holo states of AF3-NGB were approximately 1.10 and 1.13 Å, respectively, whereas those of the apo and holo states of EXP-NGB were approximately 2.04 and 1.52 Å, respectively. The average radius of gyration (Rg) values of the apo and holo states of AF3-NGB were approximately 15.44 ± 0.08 and 15.43 ± 0.08 Å, respectively, which were generally comparable to those of the apo and holo states of EXP-NGB, with average Rg values of 15.56 ± 0.15 and 15.45 ± 0.12 Å, respectively (Figure 6B). The solvent-accessible surface area (SASA) values of the apo and holo states of AF3-NGB were approximately 85.0 ± 1.835 and 85.73 ± 1.535 nm^2^, respectively, whereas those of the apo and holo states of EXP-NGB were 86.48 ± 2.40 and 85.0 ± 1.651 nm^2^, respectively (Figure 6C). The RMSF analysis revealed that both the apo and holo states of AF3-NGB exhibited lower residue-level fluctuations than those of EXP-NGB (Figure 6D). In particular, the disulfide bond region between Cys46 and Cys55 in EXP-NGB displayed significantly higher flexibility (Figure 6D). Furthermore, the heme-binding region containing Gly99 in the apo state of EXP-NGB exhibited significantly higher fluctuations than those in the other MD models, suggesting that heme binding contributes to stabilization of the heme-binding region in the holo state of EXP-NGB. Altogether, the overall structures of the apo and holo states of AF3-NGB and EXP-NGB models maintained stable folds; however, local flexibility differed based on RMSD and RMSF values.

The B-factor putty representation of the crystal structure of holo-state NGB revealed that the heme-binding region was relatively rigid, whereas the region between Cys46 and Cys55, together with the N- and C-terminal regions, exhibited relatively high flexibility (Figure 6E). In the MD simulations, the positions of Cys46 and Cys55 differed between AF3-NGB and EXP-NGB. Despite the absence of a disulfide bond between Cys46 and Cys55 in AF3-NGB, the corresponding region remained relatively rigid, and its flexibility further reduced upon heme binding (Figure 6F). Conversely, the corresponding region in EXP-NGB displayed relatively high flexibility, similar to that observed in the crystal structure of NGB (Figure 6G). These data indicated that the failure of disulfide bond formation in the AF3-predicted NGB resulted in different folding and altered molecular flexibility in the region containing Cys46 and Cys55. Meanwhile, the Cys46-Cys55 disulfide-bond region of EXP-NGB exhibited higher flexibility than the corresponding region of AF3-NGB. Because the Cys46-Cys55 disulfide bond in EXP-NGB is flanked by loop regions on both sides, these neighboring loops may contribute to the increased flexibility observed during the MD simulations. In contrast, although AF3-NGB does not contain the Cys46-Cys55 disulfide bond, this region was predicted to adopt a relatively stable local fold, which may have resulted in lower flexibility compared with EXP-NGB. These observed flexibility differences between AF3-NGB and EXP-NGB were evaluated under restrained heme-coordination conditions.

To understand the molecular flexibility of the disulfide bond and heme-binding region in the holo states of AF3-NGB and EXP-NGB, structural ensembles comprising 20 MD models were extracted from the trajectories at 199.8–200 ns, where the structures were stabilized without transient fluctuations based on the RMSD analysis. In AF3-NGB, Phe42 and Tyr44 stabilized the heme molecule, and the side chain of Tyr44 was located beneath the heme molecule with relatively low flexibility (Figure 6H). The position of Cys55 in AF3-NGB remained rigid, whereas Cys46, which was oriented toward the solvent region, displayed relatively high flexibility (Figure 6H). In EXP-NGB, the side chain of Tyr44 was located beside the heme molecule and exhibited significant flexibility (Figure 6I). The disulfide bond region formed by Cys46 and Cys55 of EXP-NGB remained relatively rigid (Figure 6I). These data suggest that the incorrect AF3-predicted model structure produces misleading interpretations of molecular flexibility and local structural dynamics.

## 3. Discussion

HBPs play a vital role in various biochemical and metabolic pathways. Understanding the molecular structures of HBPs provides both insights into their reaction mechanisms and a foundation for their rational engineering for industrial applications. When experimentally determined HBP structures are unavailable, structural modeling serves as an important alternative method for structural and functional analysis. Recent AI-based structure prediction techniques have demonstrated higher accuracy than conventional homology-based modeling methods. Therefore, to determine the reliability of AF3-predicted HBP structures for practical applications, this study investigated AF3-HBP models through structural comparisons with experimentally determined HBP structures.

The AF3-HBP structures showed reliable pTM and ipTM scores and high overall structural similarity to the experimentally determined structures of human SO-b5, Ncb5or-b5, and CYB5B. Moreover, docking analysis of the heme molecules in SO-b5, Ncb5or-b5, and CYB5B revealed that the heme molecules were generally correctly positioned within the heme-binding sites of HBPs. Although subtle movements of the heme molecules and their surrounding residues were detected, the AF3 models were overall consistent with the experimental structures. These findings suggest that AF3 can predict the overall heme-binding configurations of HBPs with relatively high accuracy, although the possible impact of training on related structural data cannot be excluded. Meanwhile, AF3 generated heme-binding models that were generally consistent with experimental structures; however, several limitations should be considered. AF3 does not allow explicit specification of the Fe oxidation state, spin state, charge state, or histidine protonation/tautomeric state. Therefore, while AF3 can successfully predict overall heme-binding modes and protein folds, caution is required when interpreting detailed heme coordination geometry and functional states of heme proteins. Experimental validation remains important for understanding the biochemical roles of heme coordination.

Although AF3-NGB also displayed high and reliable pTM scores of 0.90–0.91, its overall folding differed significantly from that of EXP-NGB due to the absence of a disulfide bond between Cys46 and Cys55. This difference affected not only the overall folding but also the positions and conformations of amino acid residues surrounding the heme-binding site. In addition, the MD simulations demonstrated that the absence of the disulfide bond affected the molecular flexibility of both the heme-binding region and the disulfide bond region. These findings suggest that AF3 predicted a structurally distinct NGB conformation lacking the disulfide bond, despite the availability of experimentally determined NGB crystal structures containing the Cys46–Cys55 disulfide bond. Hence, AF3 did not simply reproduce previously determined experimental structures but rather generated an alternative conformational model through its prediction framework. Meanwhile, because distance restraints were applied to maintain the Fe–His coordination during the simulations, the present MD results should be interpreted within the context of restrained heme-coordination conditions. Therefore, these simulations do not provide an independent assessment of the intrinsic stability of the heme-binding pocket.

The formation of disulfide bonds can be affected by the biochemical environment, such as reducing or oxidizing conditions [56,57]. Nevertheless, AF3 does not explicitly consider these environmental factors during structure prediction, which may influence the formation of disulfide bonds in the predicted models. To determine whether AF3 can accurately predict proteins containing disulfide bonds, lysozyme and thaumatin, which contain four and eight disulfide bonds in their experimental structures, respectively [58,59], were modeled using AF3. The AF3-predicted structures of both lysozyme and thaumatin successfully reproduced all disulfide bonds consistent with those observed in the experimental structures (Supplementary Figure S9). These findings suggest that the accuracy of AF3 in predicting disulfide bond formation varies depending on the structural properties of individual proteins.

Previous studies have reported that AF2 may incorrectly predict native disulfide bonds, particularly in cysteine-rich proteins and peptides, and recent reviews have emphasized the need for careful interpretation of AlphaFold models for disulfide-containing proteins [60,61,62,63]. More recently, AF3 was shown to overcome several of these limitations by correctly predicting disulfide bonds in small disulfide proteins, including cases where AF2 failed [60]. In contrast, in the present study, AF3 correctly reproduced the Cys15–Cys120 disulfide bond in NGB but failed to predict the experimentally observed Cys46–Cys55 disulfide bond. These findings suggest that although AF3 has substantially improved disulfide bond prediction, its performance may vary among different disulfide pairs. Therefore, this finding provides an example of a remaining limitation of AF3 in accurately predicting native disulfide bonds.

The formation of disulfide bonds can potentially impact not only protein folding and flexibility but also thermal stability [64,65,66,67]. MD simulation analyses of AF3-NGB and EXP-NGB clearly revealed substantial differences in the molecular flexibility of the disulfide bond region and heme-binding region between the two models. These findings indicate that inaccuracies in predicted models can result in significantly different structural and dynamic interpretations in downstream computational analyses, including MD simulations. Therefore, when predicting the structures of HBPs containing multiple cysteine residues using AF3, it is necessary to consider manual inspection of potential disulfide bond formation based on previous experimental evidence, even when the predicted models demonstrate high pTM scores. Meanwhile, only a single MD simulation was performed in this study due to computational resource limitations. Future studies involving multiple independent MD simulations may improve the statistical robustness of the observed molecular flexibility of AF3-NGB and EXP-NGB.

Although AF3 successfully docked the heme molecule into the heme-binding cavity of the HBPs, the detailed conformation and specific interactions of heme within AF3-HBPs differed significantly from those in EXP-HBPs. Heme molecules can adopt various vibrational modes, such as saddling, ruffling, doming, and breathing, and can be distorted depending on their coordination and interaction environments [68]. However, AF3 docking may not completely capture subtle heme distortions associated with functional states, indicating that experimental structural data still require an understanding of the precise molecular mechanisms of HBPs. Altogether, these findings suggest that the current version of AF3 is constrained in its ability to predict fine structural variations in heme molecules in HBPs. Nonetheless, if AF3 provides reliable heme-binding models, as found for SO-b5, Ncb5or-b5, and CYB5B, it can still reliably identify heme-binding sites and key interacting residues, providing a useful basis for protein engineering.

In the present study, the calculated volumes of the heme-binding cavities from AF3-predicted models for each HBP exhibited a wide range of variation. The heme-binding cavity volumes of EXP-Ncb5or-b5 and EXP-NGB were similar to those of their AF3-predicted counterparts, whereas the cavity volumes of EXP-SO-b5 and EXP-CYB5B differed significantly from those of their AF3-predicted counterparts. Moreover, the binding affinities between heme and HBPs, calculated from both AF3-HBP and EXP-HBP models, exhibited no significant differences. Nevertheless, despite similar binding affinities, the predicted heme positions and distortion modes differed from the experimental structures. Hence, even when the AF3-predicted HBP structures demonstrate reliable heme-binding affinities, caution is required because the precise heme geometry and functional interpretation may differ from those observed in experimentally determined structures. In addition, the Fe–His distances, cavity volumes, and apparent binding affinities measured from AF3-predicted structures should be interpreted with caution, as AF3 does not explicitly account for the iron oxidation state (ferric versus ferrous) or specific heme coordination modes.

In this study, four representative human heme-binding proteins were selected for structural comparison between AF3-predicted models and high-resolution experimental structures. Although these proteins provide useful examples for evaluating AF3 performance in heme-binding proteins, the structural diversity of human HBPs is substantially broader. Therefore, the conclusions presented here should be interpreted within the context of the selected proteins analyzed in this study. Further investigations involving additional classes of human HBPs, including cytochrome P450 proteins, CYP46A1, hemoglobin, and other structurally diverse heme-binding proteins, will be necessary to determine whether the observations reported here can be generalized to the broader human HBP family.

In addition, the present study focused on monomeric human heme-binding proteins and their heme-binding properties. More complex heme protein systems, including substrate-bound HBPs, oligomeric HBPs such as hemoglobin, membrane-associated HBPs, covalently linked HBPs, and redox-state-dependent HBPs, were not included in this analysis. In these systems, substrate binding, oligomerization, membrane interactions, or redox-dependent conformational changes may influence the heme environment and protein structure. Therefore, the conclusions of this study may not be directly applicable to these classes of heme proteins. Future studies on these systems will further clarify the capabilities and limitations of AF3 for heme-binding proteins.

## 4. Materials and Methods

### 4.1. Selection of HBPs

Crystal structures of heme-bound human HBPs were retrieved from the Protein Data Bank (PDB) [69]. To ensure accurate heme coordination geometry, high-quality structures were selected based on the following criteria: resolution < 1.5 Å, overall B-factor < 30 Å^2^, and R_free_ value < 25%. The cytochrome b5 domain of sulfite oxidase (1MJ4), the cytochrome b5 domain of NADH cytochrome b5 oxidoreductase (3LF5), cytochrome b5 type B (3NER), and neuroglobin (7VQG) were selected for this study. Detailed information on the selected human HBP structures is listed in Table S1.

### 4.2. AF3 Structure Prediction

AF3 predictions for human HBPs were performed by inputting the amino acid sequences of the selected HBPs into the AF3 server [31]. Based on the biological and functional oligomeric states, the HBPs were modeled as monomers. Heme docking to the AF3-predicted HBP structures was performed using the default parameters provided by the AF3 server. The apo and holo states of AF3-predicted HBP structures were deposited in the ZENODO repository (https://doi.org/10.5281/zenodo.20263790, accessed on 10 May 2026).

### 4.3. Molecular Dynamics Simulation

The crystal structure of NGB (PDB code: 7VQG) and the NGB model generated by AF3 were used as initial models for MD simulations. For the holo-state NGB model structures, distance restraints were applied between the heme iron atom and the coordinating histidine residues throughout the simulations to maintain the biologically relevant heme-coordination geometry. These restraints were introduced to preserve heme binding and prevent artificial release or distortion of the heme molecule during MD simulations. Protonation states of ionizable residues were assigned according to their standard states at neutral pH using the GROMACS pdb2gmx utility. All-atom MD simulations were performed using GROMACS [70] (version 2025) with the CHARMM36 [71] force field. No manual topology modification was required for the disulfide bond. The Cys46–Cys55 disulfide bond in EXP-NGB was automatically recognized during topology generation in GROMACS and maintained throughout the simulations. In contrast, no disulfide bond was detected in AF3-NGB. The model was placed in a triclinic box with a minimum distance of 10 Å from the box boundary and solvated using the TIP3P [72] water model, followed by neutralization with counterions. Energy minimization was achieved using the steepest descent algorithm until the maximum force was <1000 kJ mol^−1^ nm^−1^. The system was subsequently equilibrated under NVT conditions for 100 ps at 310 K using a V-rescale thermostat, followed by NPT equilibration for 100 ps at 1 bar using a Parrinello–Rahman barostat. Production MD simulations were performed at 310 K for 200 ns with a time step of 2 fs. The simulation systems contained approximately 20,878 and 20,899 atoms for the apo and holo states of NGB-AF3, respectively, and 21,029 and 21,050 atoms for the apo and holo states of NGB-EXP, respectively. Coordinates were saved every 2 ps, resulting in 100,000 frames for each 200 ns production simulation. Hydrogen bonds were constrained using the LINCS algorithm. Electrostatic interactions were computed using the particle mesh Ewald method, with a cutoff of 1 nm for both electrostatic and van der Waals interactions. The mdp files used for energy minimization, NVT equilibration, NPT equilibration, and production MD simulations were added to the Supplementary Materials. The average minimum distances between the protein and its periodic images were 0.85 ± 0.49 nm for the apo state of AF3-NGB, 1.14 ± 0.42 nm for the holo state of AF3-NGB, 0.97 ± 0.57 nm for the apo state of EXP-NGB, and 1.09 ± 0.44 nm for the holo state of EXP-NGB. These results indicate that the simulation box size was sufficient to prevent interactions between the protein and its periodic images throughout the simulations. Trajectory analyses included the RMSD, Rg, SASA, and RMSF values, which were calculated using built-in GROMACS tools and in-house scripts.

### 4.4. Bioinformatics Analysis

The cavity volume of the heme-binding pockets of HBPs was calculated using CB-Dock2 [73] after removal of the heme molecule. Interactions between HBPs and heme were examined using the Protein–Ligand Interaction Profiler [74] and further analyzed by manual visual inspection. The binding affinity between HBP and heme was evaluated using the PRODIGY-LIGAND web server [75]. All structural figures were generated using PyMOL (v. 2.5.10) (http://pymol.org).

## 5. Conclusions

AF3-HBP models can provide reliable overall structures of HBPs and successfully dock heme molecules into the heme-binding pockets of HBPs. Nevertheless, detailed structural features related to heme binding within the heme-binding cavities differed from those observed in the experimental structures. In particular, AF3 failed to predict the disulfide bond formation observed in NGB. These results provide valuable insights into the application of AF3 for structural analyses of HBPs and may contribute to future improvements in the AF3 algorithm.

## Data Availability

The AF3-predicted HBP structures have been deposited in Zenodo (DOI: 10.5281/zenodo.20263790).

## Supplementary Materials

The following supporting information can be downloaded at: https://www.mdpi.com/article/10.3390/ijms27146278/s1.

## Abbreviations

The following abbreviations are used in this manuscript:

HBP	Heme-binding protein
SO-b5	Cytochrome b5 domain of sulfite oxidase
Ncb5or-b5	b5 domain of NADH cytochrome b5 oxidoreductase
CYB5B	Cytochrome b5 type B
NGB	Neuroglobin
RMSD	root-mean-square deviation
Rg	radius of gyration
SASA	solvent-accessible surface area
RMSD	root-mean-square fluctuation
