# Supplementary material for: Evaluation of AlphaFold3 for Predicting Human Heme-Binding Protein Structures
Source: Int J Mol Sci. 2026 Jul 14;27(14):6278. doi: 10.3390/ijms27146278 (PMC13410086; doi:10.3390/ijms27146278)
Supplement: Supplementary file 1 [file ijms-27-06278-s001.zip › ijms-4353897-supplementary-R4.pdf]

Supplementary Data

Evaluation of AlphaFold3 for Predicting Human Heme-Binding Protein Structures

Ki Hyun Nam*

College of General Education, Kookmin University, Seoul 02707, Republic of Korea

*Correspondence: structure@kookmin.ac.kr

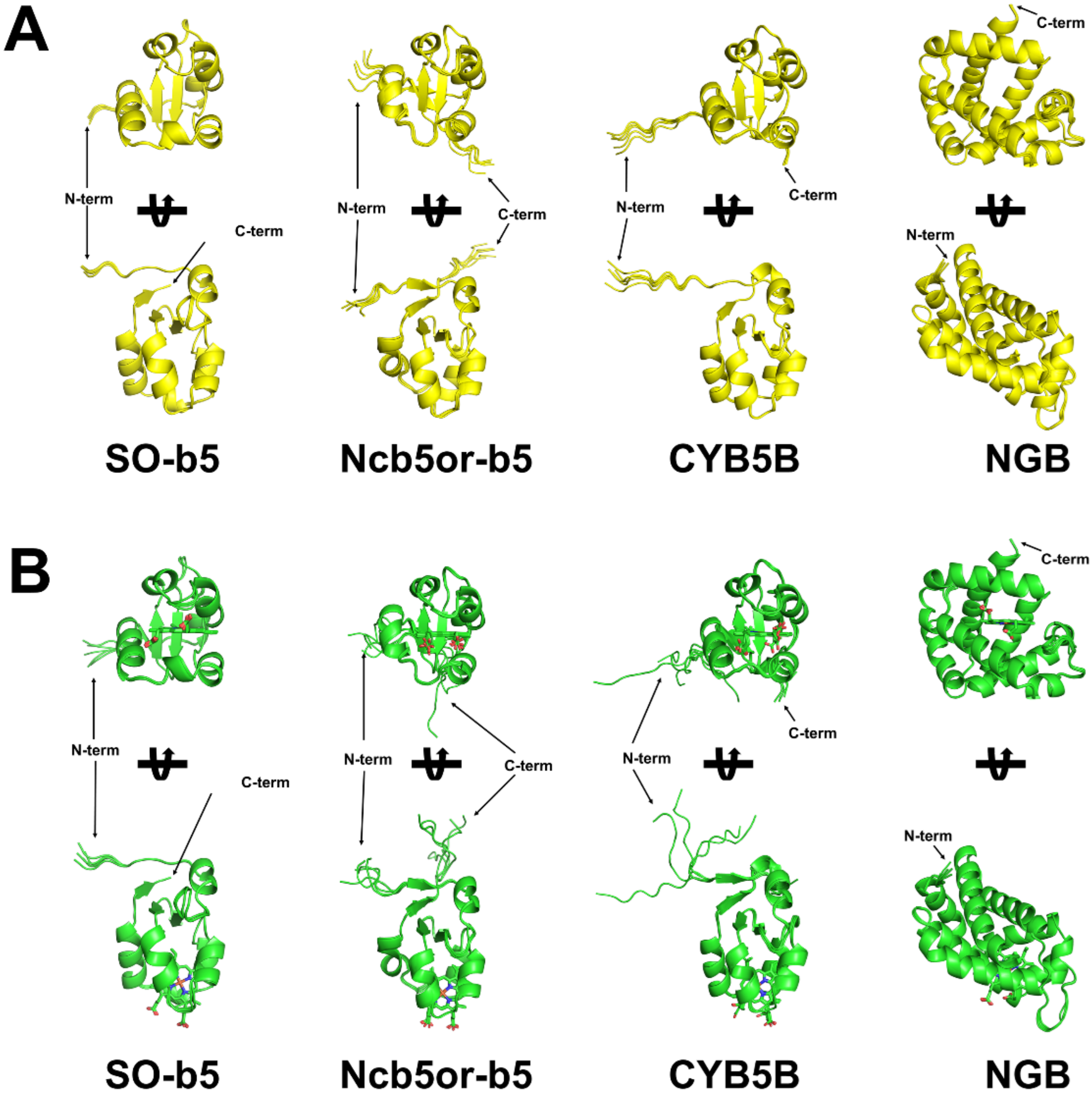

Supplementary Figure S1. Superimposition of the AF3-predicted (A) apo-state and (B) holo-state structures of SO-b5, Ncb5or-b5, CYB5B, and NGB.

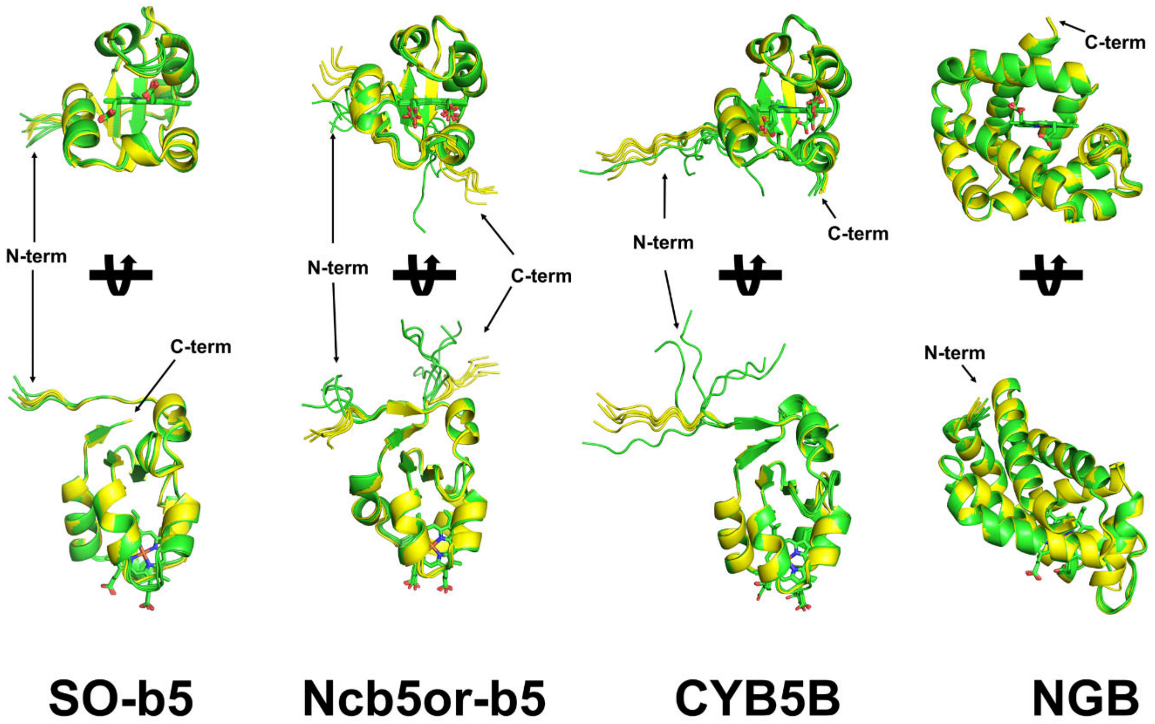

Supplementary Figure S2. Superimposition of the AF3-predicted apo-state (yellow) and holo-state (green) structures of SO-b5, Ncb5or-b5, CYB5B, and NGB.

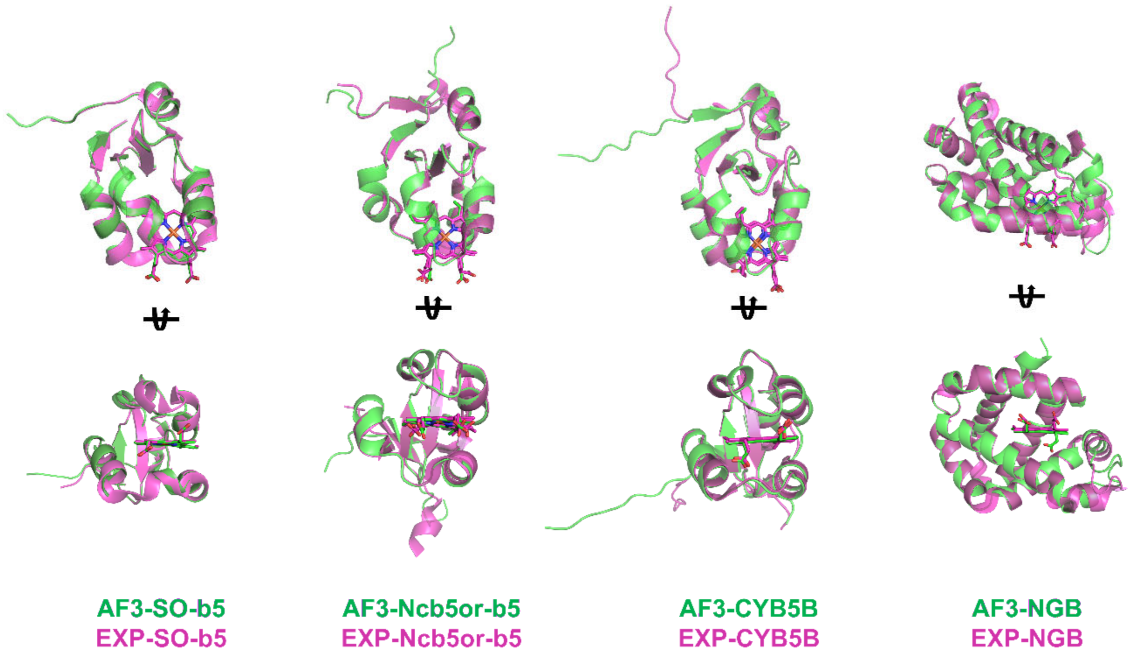

Supplementary Figure S3. Superimposition of the AF3-predicted (green) and experimental (purple) structures of SO-b5, Ncb5or-b5, CYB5B, and NGB.

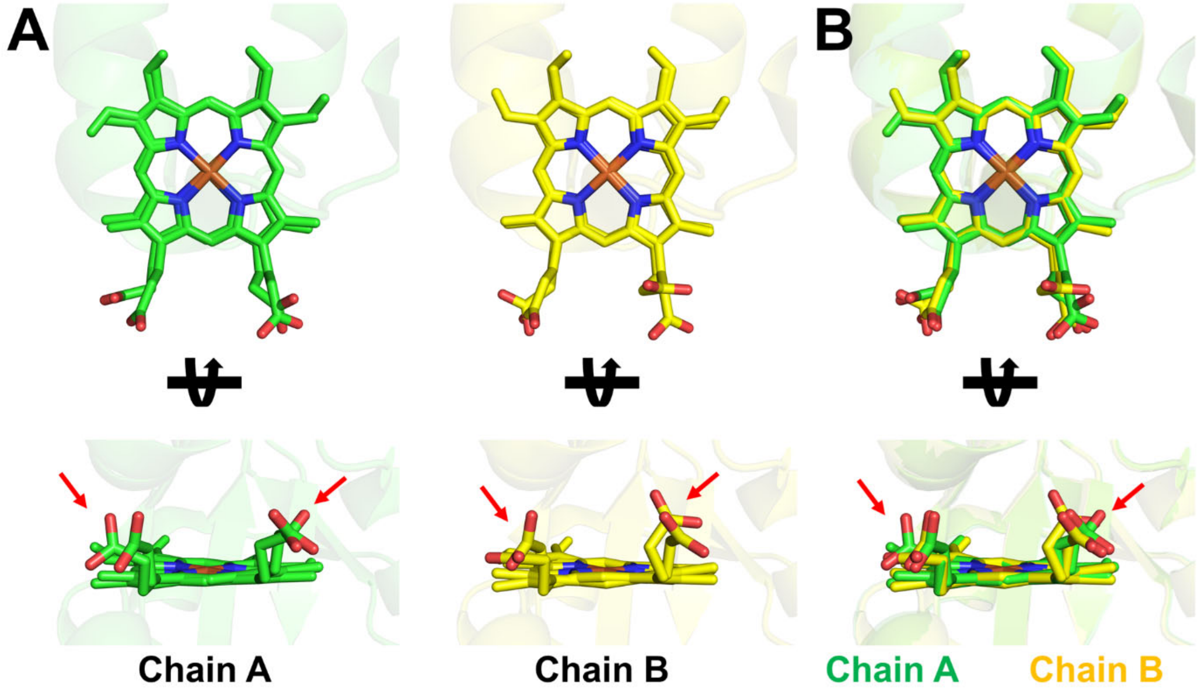

Supplementary Figure S4. Crystal structure of Ncb5or-b5 (PDB code: 3LF5). (A) Close-up view of the two different conformations of the heme molecules within the heme-binding cavity of Ncb5or-b5 from chains A and B in the asymmetric unit. (B) Superimposition of the heme molecules from chains A and B in the asymmetric unit. Conformational differences in the propionate side chains of heme are indicated by red circles.

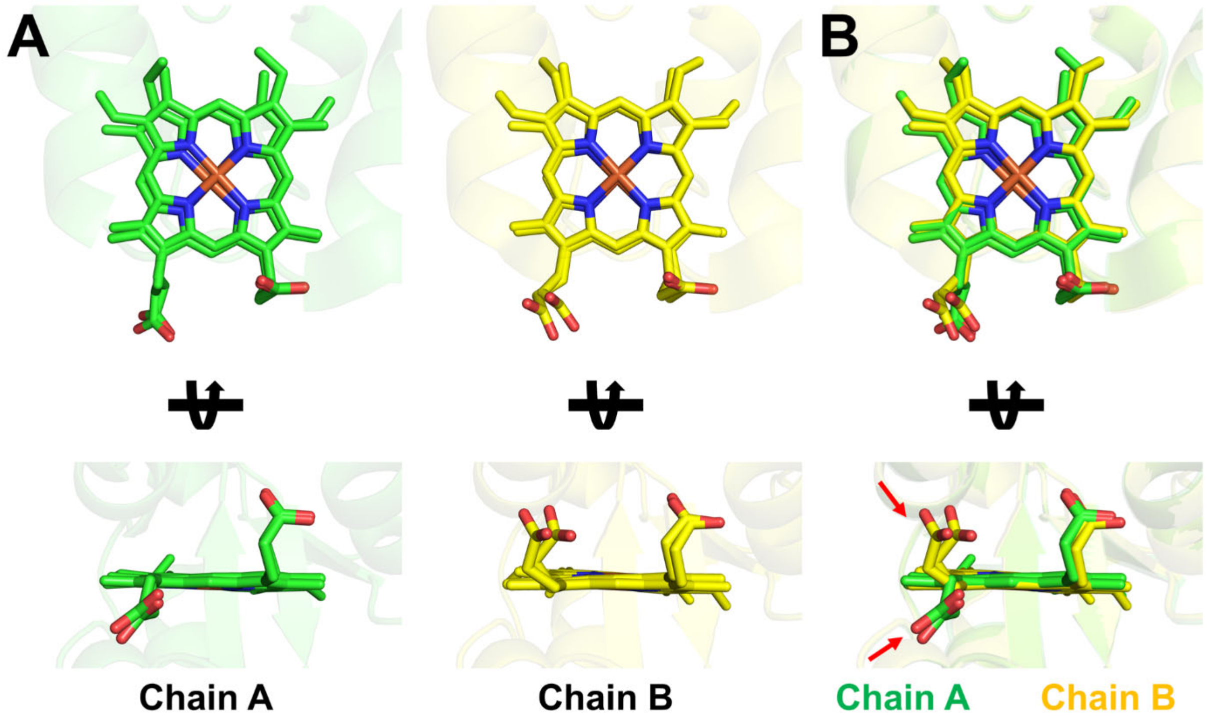

Supplementary Figure S5. Crystal structure of CYB5B (PDB code: 3LF5). (A) Close-up view of the two different conformations of the heme molecules within the heme-binding cavity of CYB5B from chains A and B in the asymmetric unit. (B) Superimposition of the heme molecules from chains A and B in the asymmetric unit. Conformational differences in the propionate side chains of heme are indicated by red circles.

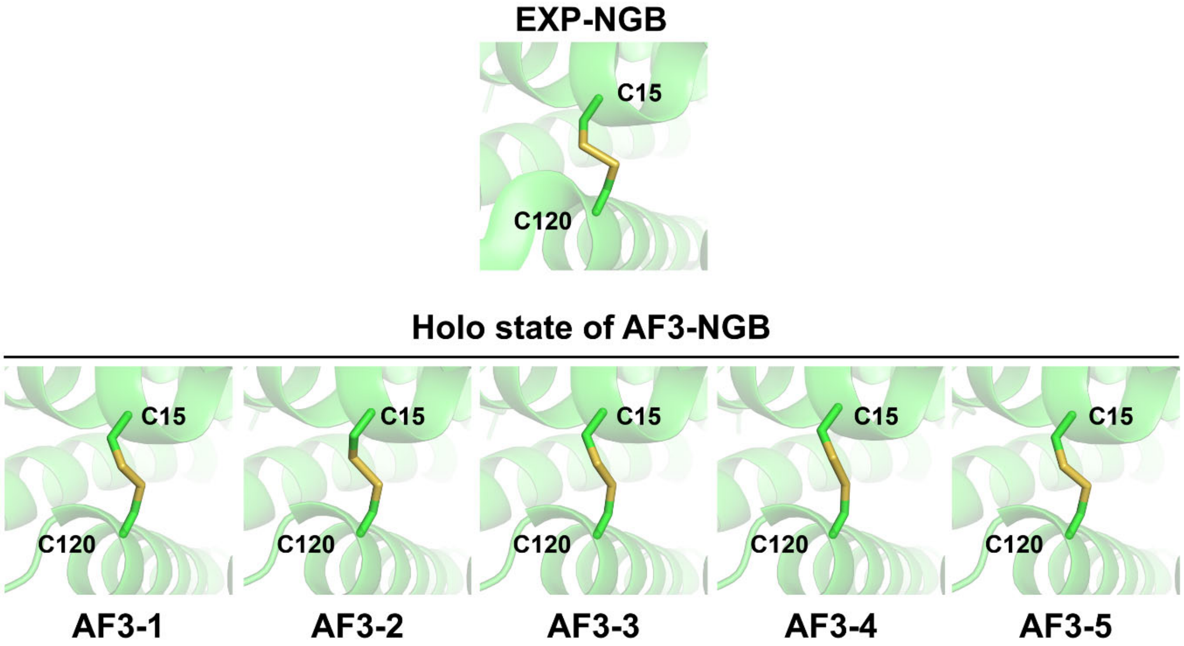

Supplementary Figure S6. Comparison of the Cys15–Cys120 disulfide bond in the experimental NGB structure (PDB ID: 7VQG) and the holo AF3-predicted NGB model.

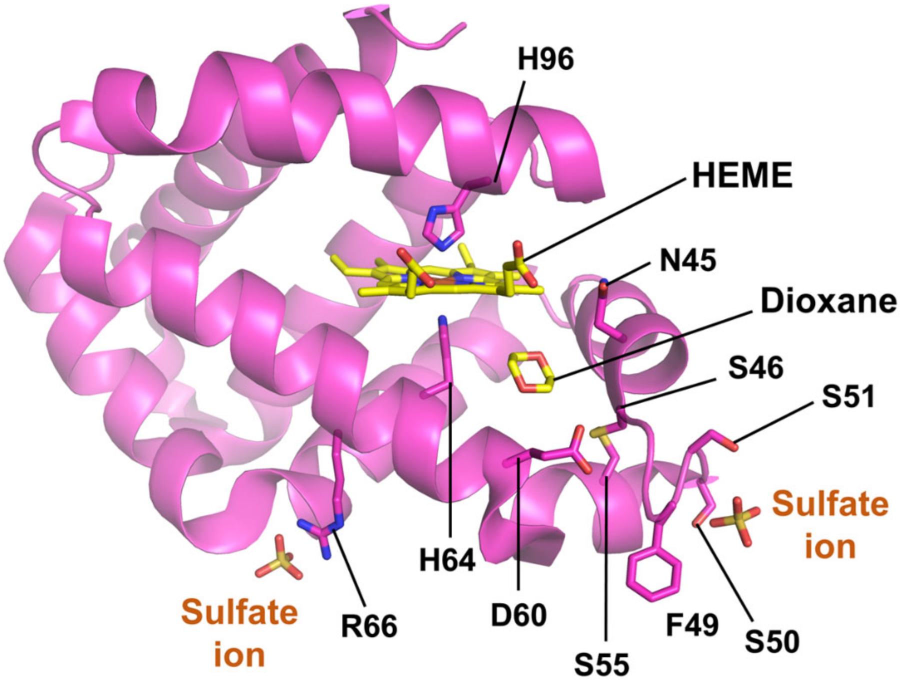

Supplementary Figure S7. Experimental structure of NGB (PDB code: 7VQG). A 1,4-dioxane molecule and a sulfate ion are located near the heme-binding site and the loop region adjacent to the disulfide-bond region, respectively.

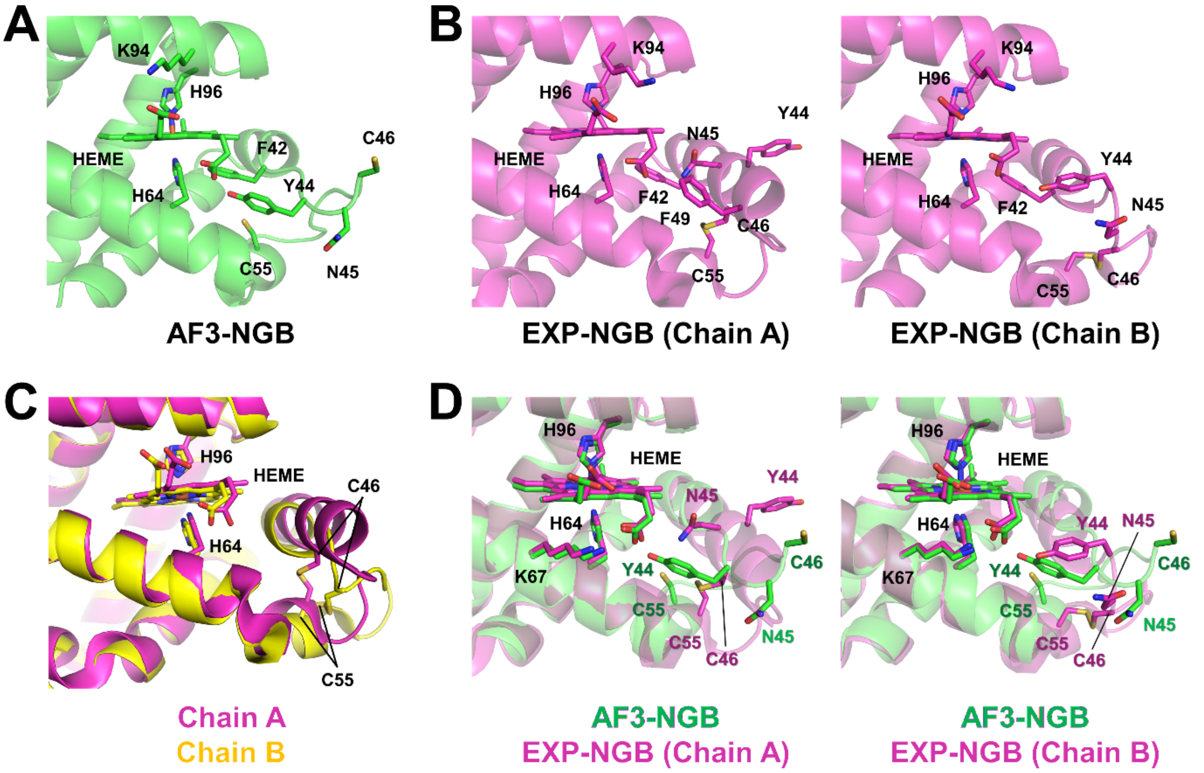

Supplementary Figure S8. Structural comparison between AF3-NGB and the experimental NGB structure (PDB code: 4MPM). Close-up views of the heme-binding site and disulfide-bond region of (A) AF3-NGB and (B) EXP-NGB. (C) Superimposition of the two EXP-NGB molecules in the asymmetric unit. (D) Superimposition of AF3-NGB (green) and EXP-NGB (magenta).

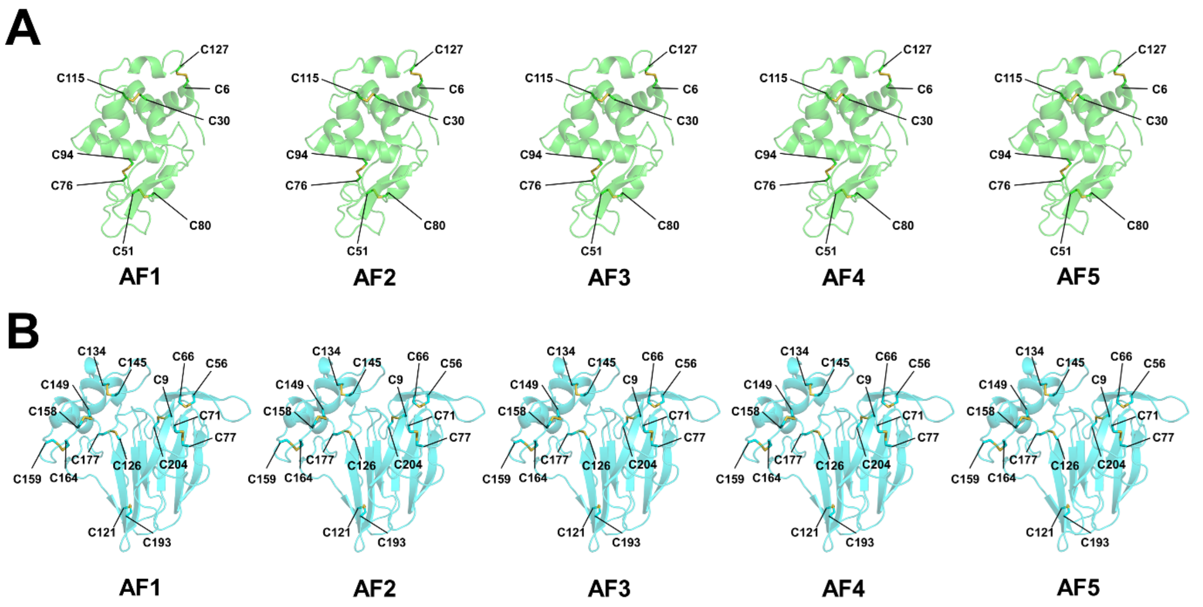

Supplementary Figure S9. Analysis of disulfide bond formation in AF3-predicted structures of (A) lysozyme and (B) thaumatin. All cysteine residues in lysozyme and thaumatin formed disulfide bonds.

Table S1. List of HBPs analyzed in this study.

HBP	PDB code	Structure name	Resolution (Å)	R_{work}/R_{free}	B-factor (Å^2)	
					Protein	HEME
SO-b5	1MJ4	Cytochrome b5 domain of sulfite oxidase	1.20	0.118/0.139	13.42	10.43
Ncb5or-b5	3LF5	b5 domain of NADH cytochrome b5 oxidoreductase	1.25	0.147/0.173	15.24	10.95
CYB5B	3NER	Cytochrome b5 type B	1.45	0.177/0.215	18.92	16.85
NGB	7VQG	Neuroglobin	1.35	0.144/0.172	22.14	19.64
